# Optofluidic platform using liquid crystals in lithium niobate microchannel

**DOI:** 10.1038/s41598-018-37351-7

**Published:** 2019-01-31

**Authors:** Silvio Bonfadini, Fabrizio Ciciulla, Luigino Criante, Annamaria Zaltron, Francesco Simoni, Victor Reshetnyak, Liana Lucchetti

**Affiliations:** 1 Dipartimento SIMAU, Università Politecnica delle Marche, Ancona, Italy; 2 Center for Nano Science and Technology, Istituto Italiano di Tecnologia, Milano, 20133 Italy; 3 Departement of Phisycs, Politecnico di Milano, Milano, 20133 Italy; 4 Dipartimento di Fisica e Astronomia G. Galilei, Università di Padova, via Marzolo 8, Padova, Italy; 5 Physics Faculty, Kyiv National Taras Shevchenko University, Prosp. Glushkova 2, Kyiv, Ukraine

## Abstract

We demonstrate the all optical control of the molecular orientation of nematic liquid crystals confined in microfluidic channels engraved in lithium niobate. Microchannels are obtained by a novel approach based on femtosecond pulse laser micromachining carried on in controlled atmosphere. The combined effect of photovoltaic and pyroelectric fields generated by light in lithium niobate crystals on the liquid crystal orientation, is reported for the first time. The total space charge field and its dependence on the incident light intensity can be controlled by changing the direction of pump light propagation through the microfluidic chip. The results reported in this manuscript demonstrate that liquid crystals and lithium niobate can efficiently be combined in microfluidic configuration, in order to push forward a novel class of optofluidic devices.

## Introduction

For many years lithium niobate (LiNbO_3_) has been among the most widely used materials in photonics^1^. The continuing popularity of LiNbO_3_ in this area originates from its highly desirable properties, such as large electro-optic, acousto-optic, piezoelectric and nonlinear optical coefficients, and its quite easy production in a single-domain state^2,3^. Moreover, this material has recently been proposed as optically active substrate for the realization of microfluidic devices^4,5^, thus paving the way for its use in the optofluidic field. On the other hand, liquid crystals (LC) are known for their sensitiveness to external stimuli, among which optical and electric fields are widely used in photonics and display applications^6,7^. The possibility of combining these two materials, thus taking advantage of the peculiarities of both, was recently demonstrated^8–11^. In particular, the light-induced control of the optical phase shift by a liquid crystal cell having LiNbO_3_ substrates^8,9^ and the generation and manipulation of defects in LC films deposited on LiNbO_3_ crystals^10,11^, have been reported. In all these papers, the key role is played by the bulk photovoltaic effect which arises in LiNbO_3_ under illumination^12^. This effect is observed in non-centrosymmetric crystals and its microscopic nature is connected to the probability of electron transition from a state with momentum k to a state of momentum k’, which is not equal to the probability of the reverse transition^13^. The resulting asymmetric momentum distribution gives rise to the appearance of a photocurrent that depends on the square of the optical field^12,13^. In open circuit conditions, the charge distribution produced by the photocurrent creates an electric field, known as photovoltaic field. Doping LiNbO_3_ with iron strongly enhances the effect^12,14^ by introducing electron donor (Fe^2+^ ions) and acceptor (Fe^3+^ ions) centres, and the photovoltaic field can become as high as 10^7^ V/m. It appears clear that the possibility to convert optical fields into electric fields offered by LiNbO_3_ conveniently combines with the LC high sensitivity to external fields.

Specifically, reference^8^ describes the possibility of using the charge separation associated to the photovoltaic effect in iron doped LiNbO_3_ (LiNbO_3_:Fe) to optically induce a static electric field able to reorient the molecular director in properly designed liquid crystal cells. In a subsequent paper^9^ a detailed analysis of the electric field generated by light irradiation in such lithium niobate-based liquid crystal cells, has been reported. The key point of these studies is the possibility of designing a new road map in the field of materials available for optofluidics, where the optically generated electric fields of LiNbO_3_:Fe can be conveniently used and configured to drive liquid crystal molecular orientation, thus allowing the fabrication of novel all optical microfluidic devices with a high degree of compactness.

With the aim of further investigating this possibility, in this work we study the response of nematic LC to the electric field optically induced in lithium niobate in microfluidic configuration. The fields coming into play are actually two, since, due to the high light intensity resulting from beam focusing, the pyroelectric field arising from laser heating cannot be neglected and its effect combines with that of the photovoltaic field creating an additional degree of freedom for controlling the LC response.

The microfluidic chip, made entirely of LiNbO_3_:Fe, consists of a rectangular microfluidic channel with two entrances (inlets and outlets) connected to the outside, formed by two symmetrical halves sealed together perfectly one above the other. The microchannel were filled with a nematic liquid crystal using a special syringe connector and the optical response of the resulting optofluidic chip was studied. Results show that the liquid crystals molecular orientation is affected by the electric fields induced in the photoactivated crystals. Although the system requires further optimization, the obtained results show several peculiar features such as the fast response time and the possibility of combining the photovoltaic and the pyroelectric fields in order to get a further control on the light induced LC reorientation when it is confined to micrometric geometries.

## Experimental Details

The two LiNbO_3_:Fe substrates have been obtained from a boule grown at the University of Padua by the Czochralski technique. The boule presents a congruent composition with a dopant concentration c_Fe_ = 18.8 × 10^18^ at/cm^3^ (0.1% mol). It was poled in air at 1200 °C, that is above the Curie temperature of the material, in order to get a single domain structure and X-Ray Diffraction measurements were realized for checking the optical quality of the sample. Then the boule was oriented along the three crystallographic axes of the material and cut in samples with the main faces perpendicular to the c-axis of lithium niobate (z-cut crystals). Finally the two LiNbO_3_:Fe substrates were polished by means of a Logitech PM5 lapping machine to achieve optical quality of the main surfaces. Then they underwent a reduction thermal treatment at 500 °C in a gas mixture of Ar (98%) +H_2_ (2%), up to a reduction degree R = Fe^2+^/Fe^3+^ of 0.07, as measured by optical absorption^15^.

The microfluidic channels have been engraved in the obtained crystals at the laboratories of the Center of NanoScience and Technology of the Instituto Italiano di Tecnologia (CNST@IIT) of Milan, through a micromachining facility consisting in an amplified 10 W femtosecond laser (Pharos, Light Conversion), providing (80–300) fs duration pulses at four wavelengths (1030 nm fundamental, 2nd, 3th and 4fh harmonic) and 1 MHz repetition rate. High precision and nanometer resolution air-bearing computer-controlled motion stages (Aerotech ABL1000), have been used to translate the samples.

**Figure 1:**
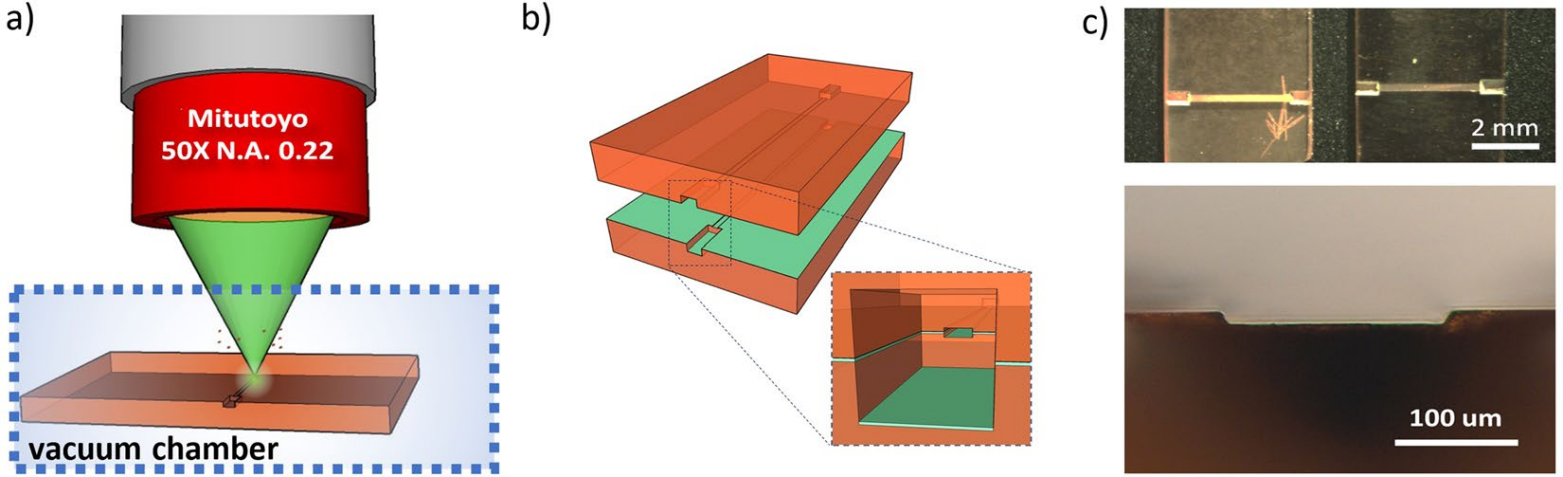
(**a**) Pictorial representation of the femtosecond micromachining writing procedure in controlled atmosphere; (**b**) sketch of the chip geometry; (**c**) optical microscope image of the chip: top and section views.

The highly localized nonlinear interaction provided by focused femtosecond laser pulses allows for sub-wavelength feature sizes smaller than the diffraction limited spot size, unlike linear absorption in long pulse laser processing. Based on the writing fluency (pulse energy/focal area), different materials properties modification can be obtained, from soft (the change of refractive index) to hard (material ablation), in 3D and even inside the bulk, simply moving the sample in relation to the spot position. This innovative chip manufacturing technique has different advantages compared to traditional photolithographic and etching techniques since it does not require a clean room and, above all, is a maskless direct-write process. In the microfluidic platforms in which quartz is the ideal material the most common method of femtosecond pulse laser microfabrication is the formation of periodic nanoplanes that are later etched by hydrofluoric (HF) acid. Although this technique has shown amazing ability to quickly fabricate precise and controllable-roughness 3D microfluidic circuits, also buried in the substrate^16^, it is not suitable for all materials. Laser assisted etching in lithium niobate has never been reported and attempts of etching LiNbO_3_:Fe crystals with HF after laser ablation, made at CNST did not give any result. Therefore direct laser ablation have been used, which normally does not guarantee low roughness of the microchannel walls if it is carried out without specific precaution. In order to reduce surface roughness, the chip was thus created in controlled atmosphere by superficially ablating its two halves in a vacuum chamber (10^−2^ Pa), as shown in Fig. 1a. Two microchannels of rectangular section (200 × 25 µm (width × height)) have been realized (Fig. 1c). The low pressure allows reducing the surface roughness of the obtained microchannels by promoting the separation of the ablated material from the crystal bulk. In this way the low size debris created by femtosecond ablation (tens of nanometers in diameter) are free to “fly” away from the unprocessed area thanks to the increase in their average free path and to the low kinetic energy possessed. The writing parameters (as well as the used objective) deeply affect the residual roughness so that the one of the side walls results almost an order of magnitude lower than that of bottom and top sides^16^. Worthy of note, surface roughness reduction in lithium niobate thin films by femtosecond micromachining in water were also proposed, showing that the ablation debris can be more efficiently removed with the assistance of water and the cavitation process triggered by the ultra-short pulse significantly reduces the size of the debris^17^. However, this “wet” process is not easy to implement, does not support high writing speeds and is not particularly suitable to long time and/or large area chip fabrications (few mm square). Furthermore, a high temperature annealing treatment of the sample is almost always necessary to achieve a satisfactory residual roughness and this may affect the LiNbO_3_:Fe properties.

Before sealing the two halves to obtain the final optofluidic chip, SiO_x_ has been deposited on the microchannel surfaces by vacuum technique in order to promote planar alignment of the LC along the channel axis. Then, the two halves were assembled paying particular attention to the alignment one above the other and sealed with a UV curable glue (Fig. 1b). The small section connecting tubes for LC inlet and outlet, fixed with the same glue conclude the manufacture.

**Figure 2:**
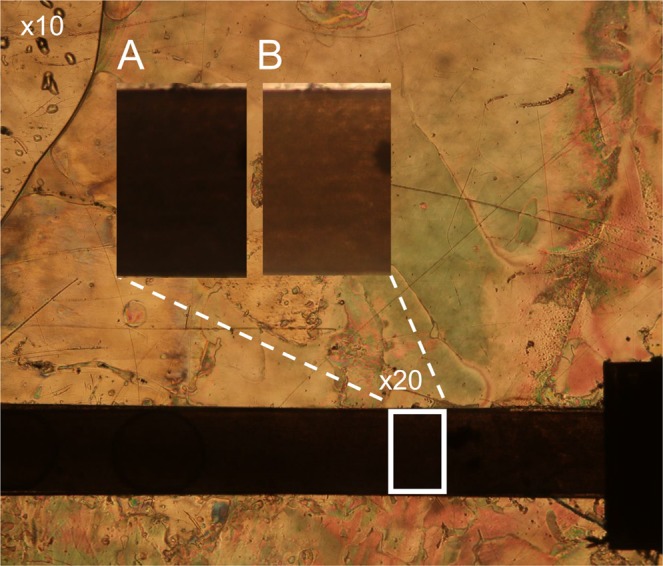
Polarizing optical microscope picture of the filled microchannel. The image is taken with the axis of the polarizer parallel to the channel axis, while keeping the analyser crossed. The two enlarged views show the increase of light transmittance typical of planar alignment occurring when the sample is rotated by 45° with respect to the polarizer axis.

The obtained microfluidic chip was then filled with the nematic eutectic mixture E7. A picture of the filled microchannel under a polarizing optical microscope is shown in Fig. 2. The image is taken with the axis of the polarizer parallel to the channel length, while keeping the analyzer crossed. The two enlarged views show the increase of light transmittance typical of planar alignment when the sample is rotated by 45° with respect to the polarizer axis. It is worth noting that the LC is at rest in the experiments here reported. A few measurements with the LC flowing in the microchannel have been performed and gave results comparable to those here described.

Pump-probe experiments were performed in the conventional geometry: the pump beam is provided by the green line of a cw Ar ion laser (λ = 514 nm) focused to a waist of 30 µm in correspondence of the microchannel central region, where it impinges at normal incidence. In this configuration, light propagates along the c axis of both LiNbO_3_:Fe crystals and, being linearly polarized in the cell plane, it does not undergo any phase shift due to the crystals birefringence. Pump light is linearly polarized parallel to the microchannel axis and its power varies in the range (40–80) mW, which leads to a pump intensity I in the range (1.5–3) × 10^3^ W/cm^2^. A mechanical shutter enabled performing irradiation cycles, which in the experiments here reported had 1 s duration and 2 s dark time separation. The probe beam originates from a low-power He-Ne laser (λ = 633 nm) focused to a waist of 20 µm and counter propagating with respect to the green pump beam. Probe polarization is linear and forms an angle of 45° with that of the pump. The probe light transmitted by the sample, orthogonally polarized with respect to the incident one, was detected by a photodiode connected to a pc. It is worth noting that the initial configuration is the one corresponding to the maximum light transmission. Each variation of the LC birefringence should lead to a decrease of the signal detected by the photodiode.

## Results and Discussion

**Figure 3:**
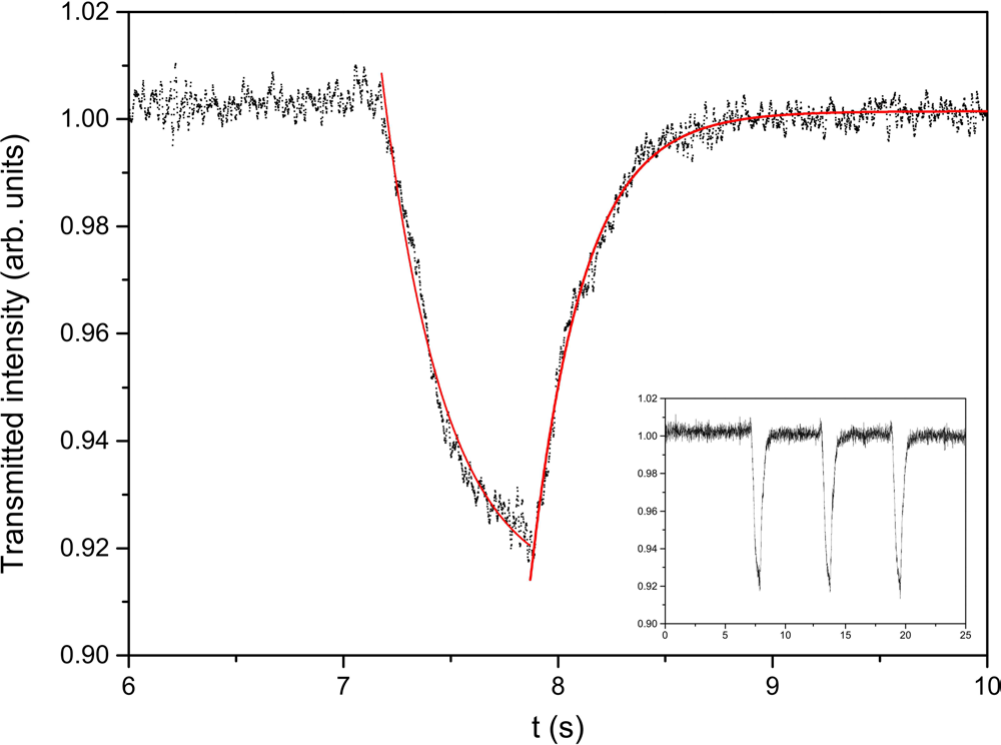
Transmitted probe signal between crossed polarizers for pump light impinging on the side of the microchip where positive charges accumulate (see text). Pump power P = 60 mW. Red lines are exponential fits giving information about the on and off times. In the case of the figure the following values are obtained: τ_ON_ = 240 ms and τ_OFF_ = 230 ms. Inset: whole curve detected for three irradiation cycles.

Pump irradiation of the LC filled microchannel leads to a decrease of the probe transmission for each value of the used power. This latter affects the amount of transmission variation, which increases by increasing the impinging pump power, and, to a lower extent, the response time. A typical example of the signal detected is shown in Fig. 3. The two exponential fits, represented by the red lines, allows to evaluate the response times. The inset reports the whole curve.

**Figure 4:**
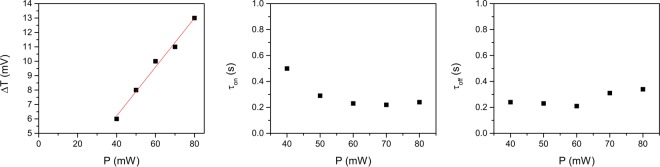
Transmission variation ΔI_T_ (**a**), on time (**b**) and off time (**c**), as a function of the pump power. Data are derived from measurements similar to those reported in Fig. 3.

From these and similar data we determined both the amplitude of the transmission variation ΔI_T_ and the response (on and off) times as a function of the pump power. Results are shown in Fig. 4. The transmission variation is linear in the pump power as shown by the linear fit in Fig. 4a. The threshold character of the induced response is also evident. Both the response times are on the order of hundreds of ms and show a weak dependence on the pump intensity. In particular, the on time is higher for low values of the incident light intensity, which could be an indication of a lower value of the stimulus responsible for the observed birefringence change. The off times shows a slight increase for higher intensity, which may be the indication of the onset of some memory effect in the light-induced modification of lithium niobate charge distribution, similarly to what happen in thermal fixing mechanisms. Indeed, measurements performed by increasing the exposure time lead to signal relaxation to values different from the unperturbed ones.

It is worth noting that measurements performed before filling the microchannel with E7 did not show any variation of the probe light transmission, thus ruling out any effect due to the birefringence of the lithium niobate substrates. Moreover, light irradiation of conventional LC planar cells with the same parameters used in the experiments described above, did not result in any birefringence variation.

A few measurements filling the microchannels with 5CB have also been performed but the obtained results, although demonstrating the possibility of modulating the LC refractive index, are not reliable due to heating of the LC generated by green light absorption in the lithium niobate substrates. Indeed the transition temperature to the isotropic state of 5CB is T_N/I_ ≅ 33 °C, and the parameters determining the LC response to external electric fields (such as refractive indexes, viscosity and dielectric properties) are quite sensitive to temperature variations close to room temperature. That is light absorption can easily lead to thermal instabilities. The use of E7 avoids these kind of troubles because of the higher transition temperature (T_N/I_ ≅ 60 °C).

**Figure 5:**
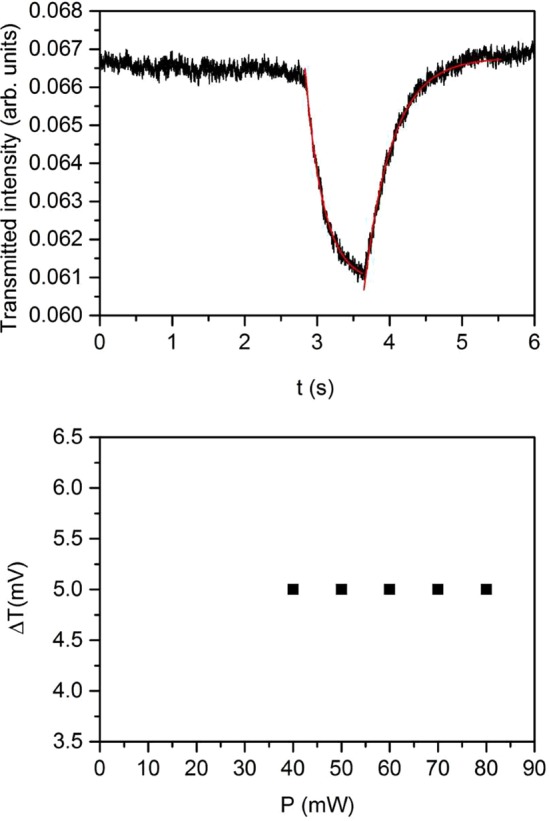
(**a**) Transmitted probe signal between crossed polarizers for pump light impinging on the side of the microchip where negative charges accumulate (see text). Pump power P = 60 mW. Red lines are exponential fits giving information about the on and off times, which are comparable to those reported in Fig. 3; (**b**) Transmission variation ΔI_T_ as a function of pump power. ΔI_T_ is lower with respect to the one reported in Fig. 4(a) and is independent on the irradiation power.

Pump probe measurements were then repeated by reverting the sample, that is by irradiating the opposite surface of the microfluidic chip so that the light wave vector and the lithium niobate c-axis change their mutual direction (see Fig. 7 further on). The results obtained in this latter configuration are shown in Fig. 5. Besides being lower than that reported in Fig. 3, the detected signal is now independent on the pump intensity (Fig. 5b). The response times are both of the same order of those reported in Fig. 4b,c.

The observed change in probe light transmission is the signature of the phase shift undergone by the light travelling along the LC filled microchannel. This on its turn is due to a change of the effective refractive index for the wave travelling through the LC cell. There are basically two possible reasons for the effective refractive index variation upon light irradiation: heating of the LiNbO_3_:Fe substrates and, consequently, of the LC in the microchannel, and director reorientation in the electric field induced by light in the LiNbO_3_:Fe substrates.

**Figure 6:**
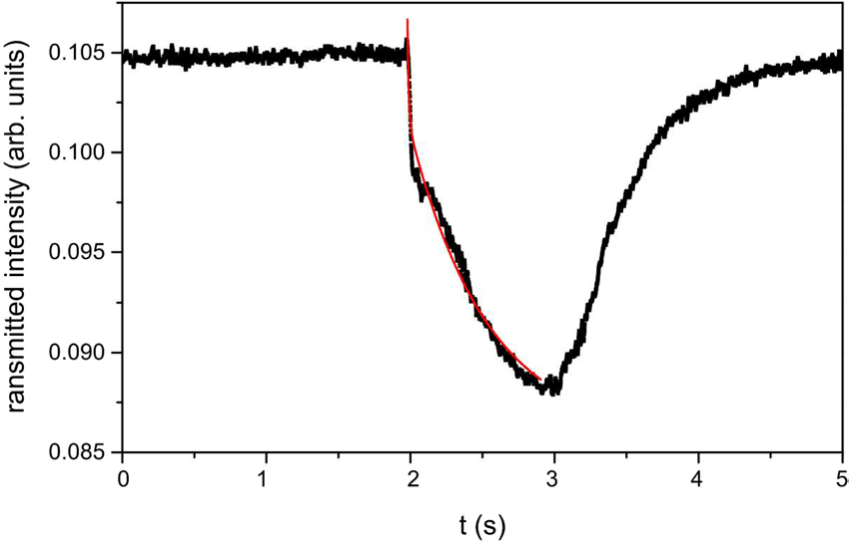
Transmitted probe signal between crossed polarizers for pump power P = 150 mW. The double exponential fit of the decreasing part of the signal is visible (red line). The two characteristic times are 9.6 ms and 300 ms.

Light-induced heating of the substrates has to be taken into account, since the wavelength of the pump beam is in the absorption band of LiNbO_3_:Fe crystals used here as optofluidic platform. Specifically, the absorption coefficient measured at λ = 514 nm for each crystal, based on the relation between incident I_0_ and transmitted I_T_ intensity through an absorbing slab of thickness d and absorption coefficient α:

1 $${I}_{T}={I}_{0}\exp (\,-\,\alpha d)$$

is 5 cm^−1^. With this value in hand it is possible to evaluate the induced temperature rise upon laser irradiation, according to the relation^18^:

2 $${\rm{\Delta }}T={(\frac{1}{{w}^{2}}+\frac{{\pi }^{2}}{{d}^{2}})}^{-1}\frac{\alpha }{K}I$$

being w the beam waist, d the thickness of the lithium niobate crystal and K the thermal conductivity. In the experimental configuration used w = 30 µm, d = 900 µm and I is in the range (1.5–3) × 10^3^ W/cm^2^. Using for K the value 10^−2^ cal/s/cm/°C^3^ valid for undoped crystals (which is expected to be lower than the one of iron doped crystals), the maximum temperature rise at steady state ranges between 2 and 4 °C. This may induce heating of the LC with a consequent change of the effective refractive index seen by the probe light travelling along the LC region. However, results shown in Fig. 5 clearly rules out LC heating as possible responsible of the observed variation of probe transmission, because this effect should not produce any asymmetric behavior. The two lithium niobate substrates that compose the microfluidic chip are identical, so the light induced heating of the LC slab is expected to be the same whatever surface is the one directly irradiated by the pump beam. The temperature rise is apparently not high enough to induce a significant variation of the LC effective refractive index. Indeed using E7, such an effect is expected to come into play at even higher intensity where the temperature rise is greater and the induced variation of the extraordinary refractive index is high enough to give rise to a detectable amount of birefringence variation. Measurements performed with a pump intensity as high as 150 mW seem to confirm this hypothesis, as shown in Fig. 6. In this case, the decreasing part of the signal requires a double exponential fit function, which produces two different characteristic times, one “fast” of the order of ten ms and one “slow” of the order of hundreds of ms. Further increase of the pump intensity confirms this behaviour. The fast part of the signal, observed just for pump intensity higher than the maximum one used in this manuscript, is likely to be due to LC heating and consequent refractive index variation.

Concerning the second possible effect, namely director reorientation under the electric field induced by light in lithium niobate, it has already been demonstrated for LC planar cells having LiNbO_3_:Fe substrates^8,9,19^. In those cases, LC reorientation was driven by the photovoltaic field generated in the two substrates by light irradiation. In the experiments discussed here, due to beam focusing required to irradiate an area inside the microchannel, the impinging intensity is higher which results in a higher temperature increase (with the values discussed above). In these conditions, the pyroelectric field can also come into play. Such a field depends on the temperature variation according to the relation^20^:

3 $${E}_{py}=-\frac{1}{{\varepsilon }_{0}{\varepsilon }_{r}}p{\rm{\Delta }}T$$

where p is the pyroelectric coefficient of the material. This parameter depends on the variation of spontaneous polarization with temperature and for congruent LiNbO_3_ crystals is p ≅ (8 × 10^−5^) C/m^2^ °C^21^. Specifically, heating due to light absorption is known to produce the so called secondary pyroelectricity^22^, an effect where the polarization is induced piezoelectrically due to the temperature induced stress and deformations of the crystal. This effect has been proved to produce macroscopic charge separation^22^, it can thus combine with the photovoltaic effect.

**Figure 7:**
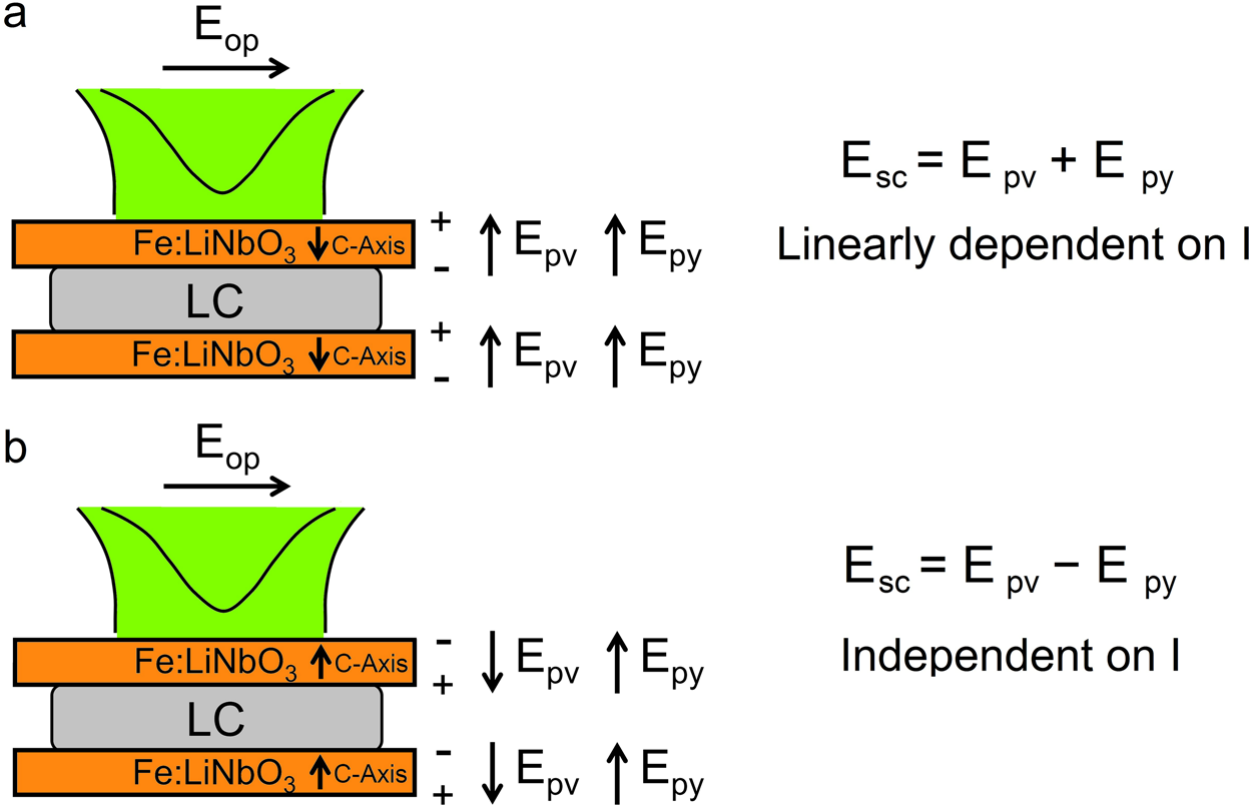
Sketch of the directions expected for the two electric fields induced by light in the LiNbO_3_ crystals, depending on the direction of light propagation. (**a**) Pump light propagating along the c-axis. This gives rise to a photovoltaic field opposite to the light wave-vector (**b**) pump light propagating in the direction opposite to the c-axis, which gives rise to a photovoltaic field along the light wave-vector. The pyroelectric field keeps its direction opposite to the photoinduced temperature gradient in both cases.

The dependence of the detected birefringence variation on the particular irradiated surface, which is evident comparing Figs 3 and 4 with Fig. 5, demonstrates that the effect is due to the actions of the electric fields induced by light irradiation on the LiNbO_3_ crystals and indicates that the two fields combine in different ways in the two different situations. In our experimental conditions both the photovoltaic field and the field due to the secondary pyroelectric effect have the direction of the c axis, which is also the direction of light propagation. Specifically, since the photovoltaic field can be parallel or antiparallel to the light wave-vector depending on which surface the beam is impinging on and assuming that the pyroelectric field is antiparallel to the temperature gradient^20^, the two fields can sum up or compensate to a certain extent depending on which is the irradiated surface. To better understand this point let us consider the sketch in Fig. 7a. Here the pump light impinges on the first crystal on the side where the positive charge develops, which gives rise to a photovoltaic field opposite to the light wave-vector. The pyroelectric field is in the same direction, since the temperature gradient is along the light wave-vector. In this situation the two fields are parallel and sum up their contributions. By reverting the sample, as in Fig. 7b, light impinges now on the surface where negative charges accumulates, thus inverting the direction of the photovoltaic field while the pyroelectric field direction does not change. The two fields are now antiparallel to each other and subtract.

Worthy of note, the primary pyroelectric effect cannot combine with the photovoltaic one in the way described, since under its action negative charges move in the +Z direction in both the situations depicted in Fig. 7, that is the primary pyroelectric field is in both cases parallel to the photovoltaic field.

The dependence of the induced response on the impinging intensity can give us a hint on which of the two fields is playing the major role in the two situations. As shown in Fig. 4a, the amount of transmission variation in the configuration shown in Fig. 7a increases linearly with the power of the pump beam, thus indicating that the field inducing the birefringence change is on its turn intensity-dependent. The pyroelectric field, being induced by temperature variation, is expected to depend linearly on the amount of light irradiation. On the other hand it is known that the photovoltaic field optically induced in iron doped LiNbO_3_ crystals is independent on light intensity for values lower than 10^7^ W/m^2^ and becomes intensity dependent for higher values of I, as predicted by the so-called “two-center model”^12^. Specifically, the photovoltaic field can be written as the sum of two terms: the field due to iron ions and the one due to polarons. This latter dominates at high intensity due to the appearance of a contribution to the current density that increases quadratically with I. Similarly, the photoconductivity presents two terms, which are proportional to I and I^2^, respectively. However, for the dopant concentration and the light intensities used in this work the second contribution is almost five orders of magnitude lower than the former one (σ (I) ∼ 10^−8^ Ω^−1^ m^−1^, σ (I^2^) ∼ 10^−14^ Ω^−1^ m^−1^), as it can be easily derived by the coefficients reported in^23^. Since the dominant term in photoconductivity is linear in I, one expects the photovoltaic field at high intensity to be composed by an intensity independent term plus a term linearly dependent on I. The different behaviour observed in the two configurations discussed, thus suggests the following scenario.

The total space charge field in the configuration shown in Fig. 7a can be written as:

4a $${E}_{sc}={E}_{PV}(I)+{E}_{PV}+{E}_{py}(I)={E}_{sc}(I)$$

Where as the total space charge in the opposite configuration (Fig. 7b) becomes:

4b $${E}_{sc}={E}_{PV}(I)+{E}_{PV}-{E}_{py}(I)={E}_{PV}$$

in the hypothesis of a total compensation of the two intensity dependent terms. Indeed, according to the two-center model^12^ the expected intensity-independent component of the photovoltaic field can be calculated to be in the range (6–8) × 10^6^ V/m, whereas the intensity-dependent component is predicted to vary from 9 × 10^5^ V/m at 40 mW to 2 × 10^6^ V/m at 80 mW, being comparable with the electric field induced by temperature gradients. This latter can be evaluated by assuming a temperature gradient in the range (2–4) × 10^3^ K/m (ΔT = (2–4) °C along a 900 µm thick crystal), to be of the order of 10^6^ V/m. Full compensation of the intensity dependent terms by means of pyroelectric effects in the configuration depicted in Fig. 7b, is thus reasonable.

Worthy of note the threshold observed in both Figs 4a and 5b, is typical of LC reorientation under the action of an external field orthogonal to the unperturbed optic axis^18^, The spatial profile of the photovoltaic field in the region in between two z-cut LiNbO_3_:Fe substrates has been calculated in^9^ and the main contribution has been found along the substrates normal. The expected result on the LC molecules is in this case a thershold splay deformation toward the homeotropic orientation. The additional contribution of the pyroelectric field does not change the thershold character of the LC response, which indicates that it also gives rise to a field orthogonal to the LC initial average molecular orientation, inducing the same deformation mentioned above.

An independent estimate for the magnitude of the electric field responsible for LC director reorientation can be obtained based on the observed characteristic time. The rise time for LC reorientation under the action of an external electric field (when the field is well above threshold) is given by^18^:

5 $${\tau }_{E}=\frac{\gamma }{{\varepsilon }_{0}{\varepsilon }_{a}{E}^{2}}$$

where γ is the LC rotational viscosity (γ the order of 0.1 Pa s for E7) and ε_a_ is the LC dielectric anisotropy. A characteristic time on a scale of 100 ms, as those reported in Figs 4 and 5, gives:

6 $$E=\sqrt{\frac{\gamma }{{\varepsilon }_{0}{\varepsilon }_{a}{\tau }_{E}}}\approx {10}^{6}V/m$$

in agreement with the values calculated above for the fields that come into play.

A final remark is required about the response times. In both the analyzed configurations the observed on and off times are smaller by at least one order of magnitude with respect to those expected for director reorientation in a 50 µm conventional LC cell^18^. The fast dynamics could indicate that the LC reorientation involves only a thin layer of the whole sample, however the reasons for such an event cannot be immediately ascribed to any particular characteristic of the analyzed system. Further investigations are in progress for clarifying this experimental observation.

## Conclusions

All optical control of the LC orientation in microfluidic channels engraved in iron-doped lithium niobate crystals has been demonstrated. Microchannels have been obtained by a novel approach based on femtosecond pulse laser micromachining realized in controlled atmosphere in order to reduce the surface roughness inside the microfluidic channel. The effect has been analyzed in details and several peculiar features have been highlighted. The first evidence of the effect of the secondary pyroelectric field induced in LiNbO_3_ because of laser heating, on LC reorientation has been reported. Specifically, LC director reorientation has been demonstrated to be due to a combination of photovoltaic and pyroelectric fields, which give rise to a total space charge field whose effect on LC molecular orientation and pump intensity dependence can be controlled by changing the directly irradiated surface. This behavior appears to be interesting for optofluidic applications, where the development of active or passive optical devices based on a proper material is currently the subject of wide investigation.

Due to the peculiarity of the optically induced space charge fields, the LC molecular reorientation develops following a dynamic which is faster than the one characterizing the LC response in conventional cells.
